# Inhibitory effect of esculentoside A on prostaglandin E_2_ production from murine peritoneal macrophages and rabbit synovial cells in vitro

**DOI:** 10.1080/09629359791884

**Published:** 1997-02

**Authors:** H-B. Wang, J. Fang, Q-Y. Zheng

**Affiliations:** 1 Department of Pharmacology College of Pharmacy Second Military Medical University Shanghai 200433 China

## Abstract

Esculentoside A (EsA) is a saponin isolated from the roots of *Phytolacca esculenta*. Previous experiments have shown that it has strong antiinflammatory effects. To investigate the mechanism of anti-inflammatory effects of *esculentoside* A (EsA),[^3^H] arachidonic acid (AA) prelabelled murine macrophage and radioimmunoassay were used to test the effect of EsA on the total release of AA and prostaglandin E_2_ in culture supernatants. The results showed that EsA had no significant effect on the total release of AA from murine macrophages. EsA (2.5-10 μmol/l), from unstimulated murine peritoneal macrophages and rabbit synovial cells, could decrease the production of prostaglandin E_2_. In A_23187_ and LPS-treated macrophages and synovial cells, EsA (10 μmol/l) could significantly decrease the prostaglandin E_2_ production. These results confirmed that EsA exerted an inhibitory effect on prostaglandin E_2_ production from murine macrophages and rabbit synovial cells.


Mediators of Inflammation, **6**, 22–24 (1997)

### Inhibitory effect of esculentoside A on prostaglandin $E_2$ production from murine peritoneal macrophages and rabbit synovial cells in vitro

H-B. Wang[CA], J. Fang and Q-Y. Zheng

Department of Pharmacology, College of Pharmacy, Second Military Medical University, Shanghai 200433, China

[CA]Corresponding Author
Fax: (+86) 2165 493951

**ESCULENTOSIDE A (EsA) is a saponin isolated from the roots of *Phytolacca esculenta*. Previous experiments have shown that it has strong anti-inflammatory effects. To investigate the mechanism of anti-inflammatory effects of *esculentoside* A (EsA), [$^3$H] arachidonic acid (AA) prelabelled murine macrophage and radioimmunoassay were used to test the effect of EsA on the total release of AA and prostaglandin $E_2$ in culture supernatants. The results showed that EsA had no significant effect on the total release of AA from murine macrophages. EsA (2.5–10 μmol/l), from unstimulated murine peritoneal macrophages and rabbit synovial cells, could decrease the production of prostaglandin $E_2$. In $A_{23187}$ and LPS-treated macrophages and synovial cells, EsA (10 μmol/l) could significantly decrease the prostaglandin $E_2$ production. These results confirmed that EsA exerted an inhibitory effect on prostaglandin $E_2$ production from murine macrophages and rabbit synovial cells.**

**Key words:** Arachidonic acid, Cultured cells, *Esculentoside* A, Peritoneal macrophages, Prostaglandin $E_2$, Synovial cells

### Introduction

*Esculentoside* A (EsA) is a saponin isolated from the root of *Phytolacca esculenta*, and was identified as 3-O-[β-D-glucopyranosyl-(1-4)-β-D-xylopyranosyl] phytolaccagenin. The structure of this compound is shown in Fig. 1. Previous experiments have shown that it has strong anti-inflammatory effects,[1] significantly decreasing the production of tumour necrosis factor (TNF) from LPS stimulated murine macrophages[2] and platelet activating factor (PAF) from $A_{23187}$ stimulated rat macrophages.[3] EsA also inhibited IL-1 production and phagocytic activity in murine macrophages.[4] Prostaglandin $E_2$ was an important inflammatory mediator and was found at the site of inflammation. $PGE_2$ caused vasodilatation leading to redness.[5] It has been implicated in the angiogenesis required for the spread of the pannus in the synovial hyperplasia component of rheumatoid arthritis.[6] $PGE_2$ is an arachidonic acid (AA) metabolite. In order to study the mechanism of the anti-inflammatory effect of EsA, this paper studied the effect of EsA on AA release from macrophages and the production of prostaglandin $E_2$ from murine peritoneal macrophages and rabbit synovial cells.

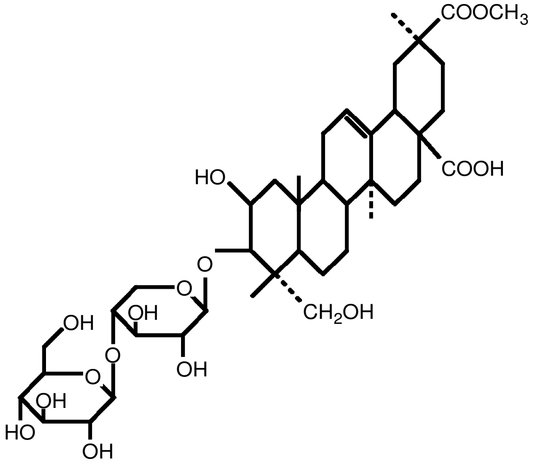

FIG. 1. The structure of esculentoside A.

### Materials and Methods

### Reagents

RPMI-1640, MEM, lipopolysaccharides (*Escherichia coli* 055:B5), calcimycin (ionophores, $A_{23187}$), zymosan, trypsin were purchased from Sigma (USA), [5,6,8,9,11,12,14,15$^3$H]arachidonic acid (8029 GBq/mmol) was purchased from Amersham. $PGE_2$ radio-immunoassay (RIA) kit was obtained from the Chinese Academy of

© 1997 Rapid Science Publishers

Medical Sciences. EsA was kindly provided by Dr Y. H. Yi (Department of Phytochemistry, College of Pharmacy, Second Military Medical University). Thioglycolate was supplied by the Shanghai Biological Research Institute.

### Preparation of murine macrophages

Thioglycolate medium (1 ml, 3%) was injected i.p. into the $C_{57}$ BL/6 mice. Four days later the cells in the peritoneal cavity were harvested with D-Hank's solution, washed twice in RPMI-640 and adhered for 2 h at 37°C in a culture bottle in a $CO_2$ incubator. The nonadherent cells were decanted and the remaining adherent cells were digested with trypsin (0.25%) for about 3–4 min. RPMI-1640 containing 10% FCS was added to the culture bottle and the cell suspension was adjusted to $10^6$ ml with RPMI-1640 containing 10% FCS and disposed at 1 ml/well in 24-well plates.

### Rabbit synovial cells culture

Synovial cells were prepared from a rabbit (weighing 2500–3000 g). Briefly, the rabbit was killed by bleeding. Synovium was taken out under ascetic conditions and cut in 1–2 $mm^3$. The synovium was directly adhered on to the bottle, MEM (including 20% FCS, 200 μg/ml glutamin) was added to the bottle. The medium was refreshed after 2–3 days. The synovium was taken out when the synovial cells confluenced.

### [$^3$H]arachidonic acid uptake by murine macrophages

The experiment was carried out as previously reported.[7] Briefly, $10^7$ macrophages in 1 ml medium (containing 10% FCS) were added to 35 mm culture dishes for 2 h. Nonadherent cells were washed away by D-Hank's solution. [$^3$H]AA, 18500 Bq in 1 ml RPMI-1640 were added to each well for 4 h. The supernatant was decanted and the cells were washed twice. Zymosan, 400 μg/ml was added to each well after EsA in 1 ml RPMI-1640 at different concentrations was co-cultured with macrophages for 20 min. The supernatants were collected at 2, 5 and 15 h and radioactivity was counted in a β-scintillator.

### Measurement of prostaglandin $E_2$

The experiment was carried out as in Ref. 6. The rabbit's synovial cells ($2 \times 10^5$/ml) and murine peritoneal macrophages ($1 \times 10^6$/ml) in 1 ml medium (MEM containing 10% FCS) was seeded in wells respectively and incubated for 24 h. The supernatants were decanted and the cells were washed with MEM three times. $A_{23187}$ and LPS were added in the presence and absence of EsA. After 24 h incubation the supernatants were adjusted to pH 3.5 with 10% HCOOH and extracted with ethylacetate twice (2 ml each time). The organic section was evaporated and the residual was reconstituted with 200 μl RIA assay buffer. The $PGE_2$ content was tested and expressed as ng/$2 \times 10^5$ synovial cells and ng/$10^6$ macrophages respectively.

### Statistics

Each experiment was carried out three times and the results presented here were representative of the three experiments. The same tendencies occurred in the parallel experiments. The results were expressed as the arithmetic mean ± SEM. The differences between the control group and treatment groups were analysed by Student's *t*-test; $P < 0.05$ was regarded as significant.

### Results

### Total release of AA from murine macrophages

EsA (2.5–10 μmol/l) had no significant effect on total release of AA from zymosan (400 μg/ml) treated murine macrophages (Fig. 2).

### $PGE_2$ production from murine peritoneal macrophages

EsA (2.5–10 μmol/l) inhibited $PGE_2$ production from unstimulated murine peritoneal macrophages. In $A_{23187}$ stimulated murine peritoneal

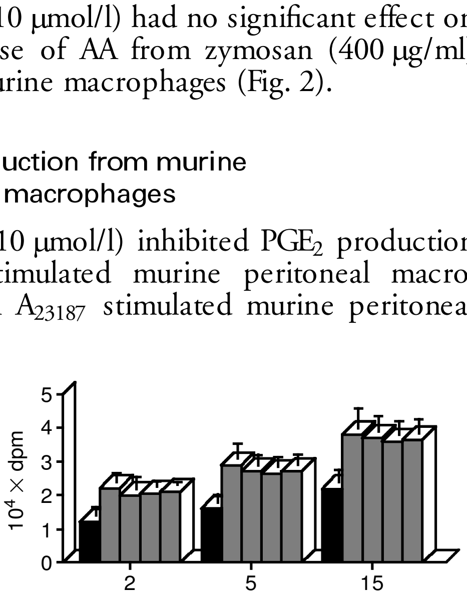

| | 2 | 5 | 15 |
|---|---|---|---|
| 0 | 1.2 | 1.6 | 2.2 |
| zymosan | 2.2 | 2.9 | 3.8 |
| EsA 2.5 | 2 | 2.7 | 3.7 |
| EsA 5.0 | 2.05 | 2.65 | 3.62 |
| EsA 10 | 2.1 | 2.72 | 3.66 |

time/h

FIG. 2. Effect of EsA on total release of AA from [$^3$H] AA prelabelled murine macrophages induced by zymosan (400 μg/ml). n = 4, mean ± SEM.

macrophages, EsA (5–10 μmol/l) could significantly suppress the $PGE_2$ production; in LPS-treated groups EsA (10 μmol/l) could significantly inhibit $PGE_2$ production (Fig. 3).

### $PGE_2$ production from rabbit synovial cells

The same results as for murine peritoneal macrophages were observed in rabbit synovial cells. EsA (2.5–10 μmol/l) inhibited $PGE_2$ production from unstimulated rabbit synovial cells. In $A_{23187}$ and LPS-treated rabbit synovial cells, EsA (10 μmol/l) could significantly suppress the $PGE_2$ production (Fig. 4).

### Discussion

The present study demonstrated that EsA suppressed the production of $PGE_2$ from murine peritoneal macrophages and rabbit synovial cells. EsA at lower concentrations could inhibit $PGE_2$ production from unstimulated macrophages and synovial cells. In LPS and $A_{23187}$ treated groups, the inhibitory concentration reached 10 μmol/l. That is to say, the inhibitory concentration of EsA on $PGE_2$ production in stimulated cells is higher than that in unstimulated cells. Prostaglandins are products of oxidation of arachidonic acid. The cyclooxygenase (COX) enzyme is the first dedicated enzyme in prostaglandin synthesis. At present, there appear to be at least two COX isozymes: a constitutive enzyme denoted COX-I which is responsible for the physiological synthesis of prostaglandins in tissue, and an inducible form termed COX-II which appears to be the major enzyme responsible for inflammatory prostaglandin synthesis. Our studies in the present paper have shown that EsA could inhibit both physiological and inflammatory prostaglandins' production from macrophages and synovial cells. $PGE_2$ is a metabolite of AA. We assumed that EsA inhibited $PGE_2$ production through its inhibitory effect on the release of AA. The results in Fig. 2 confirmed that EsA had no significant effect on the release of AA. Previous experiments proved that released AA could be re-uptake by cell membrane.[8] In the present research paper, our experiments confirmed that EsA had no effect on AA release in [$^3$H]AA prelabelled murine macrophages. We did not know whether EsA could affect AA uptake by cell membrane or not. Further experiments about the effect of EsA on AA uptake by cell membrane, activity of cyclooxygenase, lipoxygenase or other pathways are needed to clarify the anti-inflammatory mechanism of EsA.

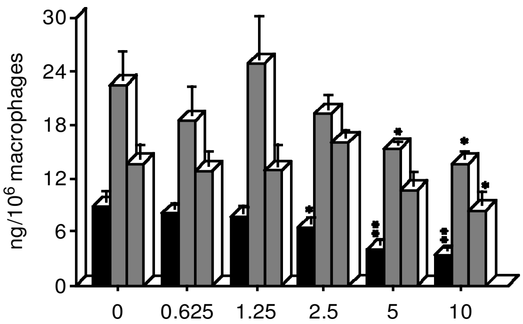

| | 0 | 0.625 | 1.25 | 2.5 | 5 | 10 |
|---|---|---|---|---|---|---|
| untreated | 8.8 | 8 | 7.6 | 6.4 | 4 | 3.4 |
| A23187 | 22.4 | 18.4 | 24.8 | 19.2 | 15.2 | 13.6 |
| LPS | 13.6 | 12.8 | 12.8 | 16 | 10.6 | 8.4 |

μmol/l

FIG. 3. Effect of EsA on the production of $PGE_2$ (ng) from $10^6$ macrophages on various states. n = 4, mean ± SEM, *$P < 0.05$, **$P < 0.01$ vs control group.

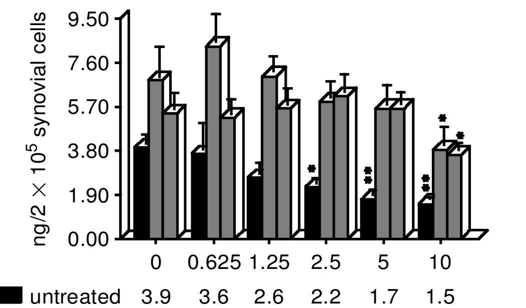

| | 0 | 0.625 | 1.25 | 2.5 | 5 | 10 |
|---|---|---|---|---|---|---|
| untreated | 3.9 | 3.6 | 2.6 | 2.2 | 1.7 | 1.5 |
| A23187 | 6.8 | 8.3 | 7 | 5.9 | 5.6 | 3.8 |
| LPS | 5.4 | 5.2 | 5.6 | 6.1 | 5.6 | 3.6 |

μmol/l

FIG. 4. Effect of esculentoside A on the production of $PGE_2$ (ng) from rabbit synovial cells ($2 \times 10^5$) on various states. $n = 4$, mean ± SEM, *$P < 0.05$, **$P < 0.01$ vs control group.

### References

1. Zheng QY, Mai K, Pan XF. Antiinflammatory effects of *esculentoside* A. *Chin J Pharmacol Toxicol* 1992; **6**: 221–224.
2. Fang J, Zheng QY, Wang HB, Yi YH. Effects of *esculentoside* A on tumor necrosis factor production by mice peritoneal macrophage. *Mediators of Inflammation* 1992; **1**: 375–377.
3. Fang J, Zheng QY. Inhibitory effects of *esculentoside* A on platelet activating factor released from calcimycin induced rat peritoneal macrophages. *Acta Pharmaceutic Sin* 1991; **26**: 721–724.
4. Ju DW, Zheng QY, Wang HB, Fang J. Effect of *esculentoside* A on immune function in mice. *Acta Pharmaceutic Sin* 1994; **29**: 252–255.
5. Robinson DW. Eicosanoids in human inflammation. In: Hedqvist P, Kalden JR, Muller-Peddinghaus R, Robinson DR, eds. *Trends in RA-Research*. Basel: EULAR, 1991; 195–198.
6. Colville-Nash PR, Scott DL. Angiogenesis and rheumatoid arthritis: pathogenic and therapeutic implications. *Ann Rheum Dis* 1992; **51**: 919–925.
7. Mai K, Yue TL. Inhibitory effect of 654-2 on release of PGs and LTs from lipopolysaccharides stimulated murine macrophages. *Sci Bull (China)* 1989; **5**: 387–390.
8. Goppelt-Struebe M. Control of prostanoid synthesis: role of reincorporation of released precursor fatty acids. *Prostaglandins* 1986; **32**: 377–385.

**Received 28 August 1996;**
**accepted in revised form 28 October 1996**
